# Condylar motion analysis: a controlled, blinded clinical study on the interindividual reproducibility of standardized evaluation of computer-recorded condylar movements

**DOI:** 10.1038/s41598-023-37139-4

**Published:** 2023-07-20

**Authors:** M. Oliver Ahlers, Tim Petersen, Lukasz Katzer, Holger A. Jakstat, Jakob C. Roehl, Jens C. Türp

**Affiliations:** 1 Department of Prosthetic Dentistry, Center for Dental and Oral Medicine, University Hospital Hamburg-Eppendorf, University of Hamburg, Martinistr. 52, 20251 Hamburg, Germany; 2 CMD Center Hamburg-Eppendorf, Falkenried 88, 20251 Hamburg, Germany; 3 Department of Prosthetic Dentistry, Dental Materials and Special Care, Center for Dental and Oral Medicine, University of Leipzig, Liebigstraße 10-12, Haus 1, 04103 Leipzig, Germany; 4 Department of Oral and Maxillofacial Surgery, Head and Neurocenter, University Hospital Hamburg-Eppendorf, University of Hamburg, Martinistr. 52, 20251 Hamburg, Germany; 5 Division Temporomandibular Disorders and Orofacial Pain, Department of Oral Health and Medicine, University Center for Dental Medicine Basel UZB, University of Basel, Mattenstrasse 40, 4058 Basel, Switzerland

**Keywords:** Oral manifestations, Classification and taxonomy, Medical research

## Abstract

The present study investigated to what extent a systematic evaluation of electronic condylar motion recordings leads to reproducible results in different examiners. The study was based on the anonymized condylar motion recordings of 20 patients (Cadiax compact II system). These were recruited consecutively from the examinations in a center specializing in diagnosing and managing temporomandibular disorders (TMD). Four trained practitioners independently evaluated the identical movement recordings of all patients after calibration. The evaluation was based on the previously published evaluation system. The results were recorded digitally in a database. The findings were then compared, and the matching values were determined (Fleiss' Kappa). The evaluation, according to Fleiss' Kappa, showed that the consistency of the assessment of the findings among the examiners is excellent (mean value 0.88, p < 0.00001). The study shows that calibrated dentists achieved reproducible results using this evaluation system and computer-assisted reporting. Good reproducibility confirms the reliability of systematic evaluation of clinical motion analysis. The ambiguities uncovered and eliminated in the study should avoid misunderstandings in the future. Both factors establish the prerequisites for applying condylar motion analysis in clinical practice.

## Introduction

Methods for recording condylar/mandibular movements pursue the goal of using the information obtained to reproduce these movements as accurately as possible in mechanical or virtual articulators. This “articulator programming” allows individual reproduction of static and dynamic occlusion for the analog or digital design and fabrication of removable dentures and fixed restorations, which is the main indication for condylar tracings. The rationale for using motion data for articulator settings is based on the consequence that even relatively small changes in movement trajectories can significantly affect the design of the occlusal surfaces^1^.

**Figure 1:**
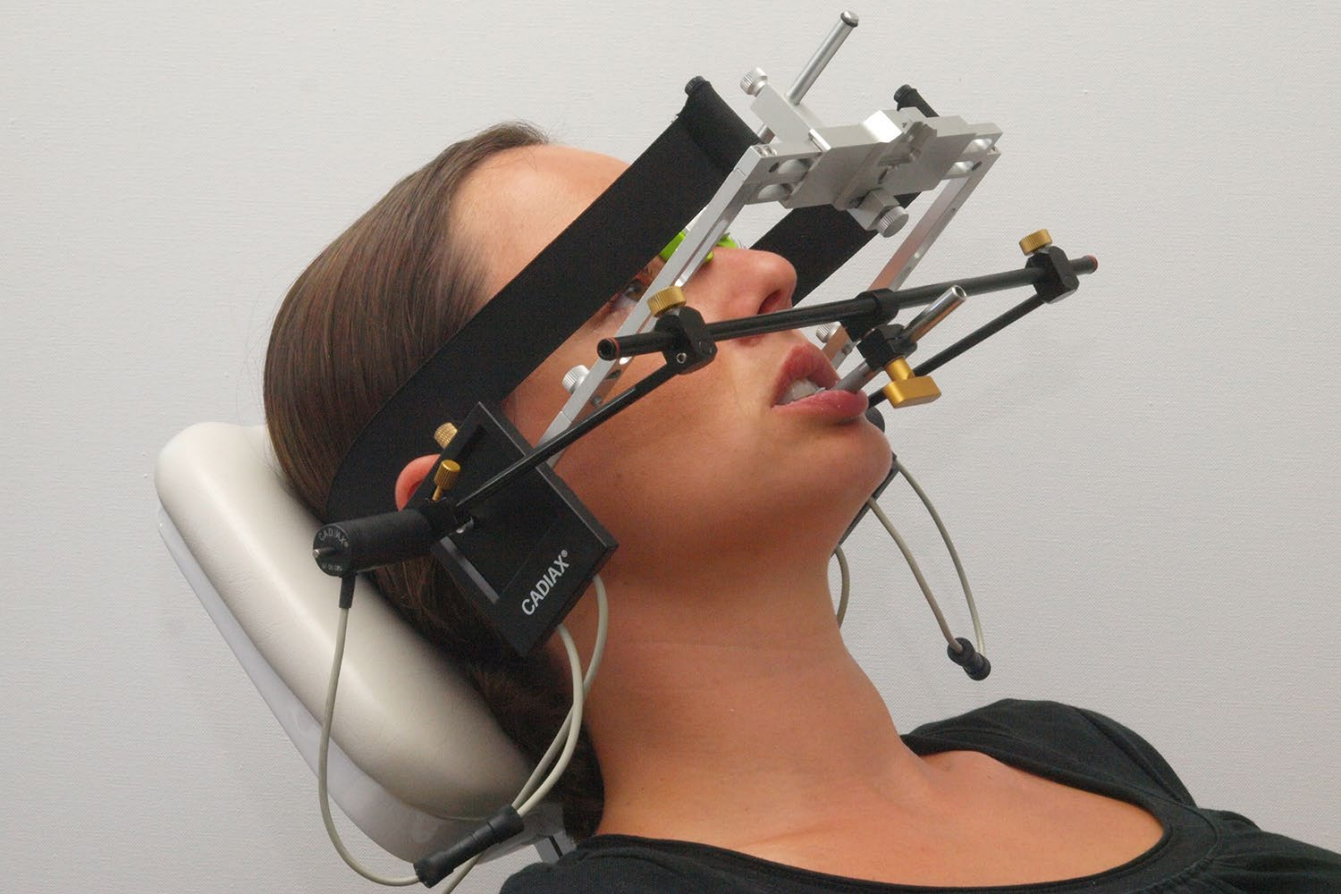
Electronic motion recording system used, which is based on the voltage division method (Gamma Cadiax Compact 2 in conjunction with Amann Girrbach Artex Facebow, photo: Ahlers).

**Figure 2:**
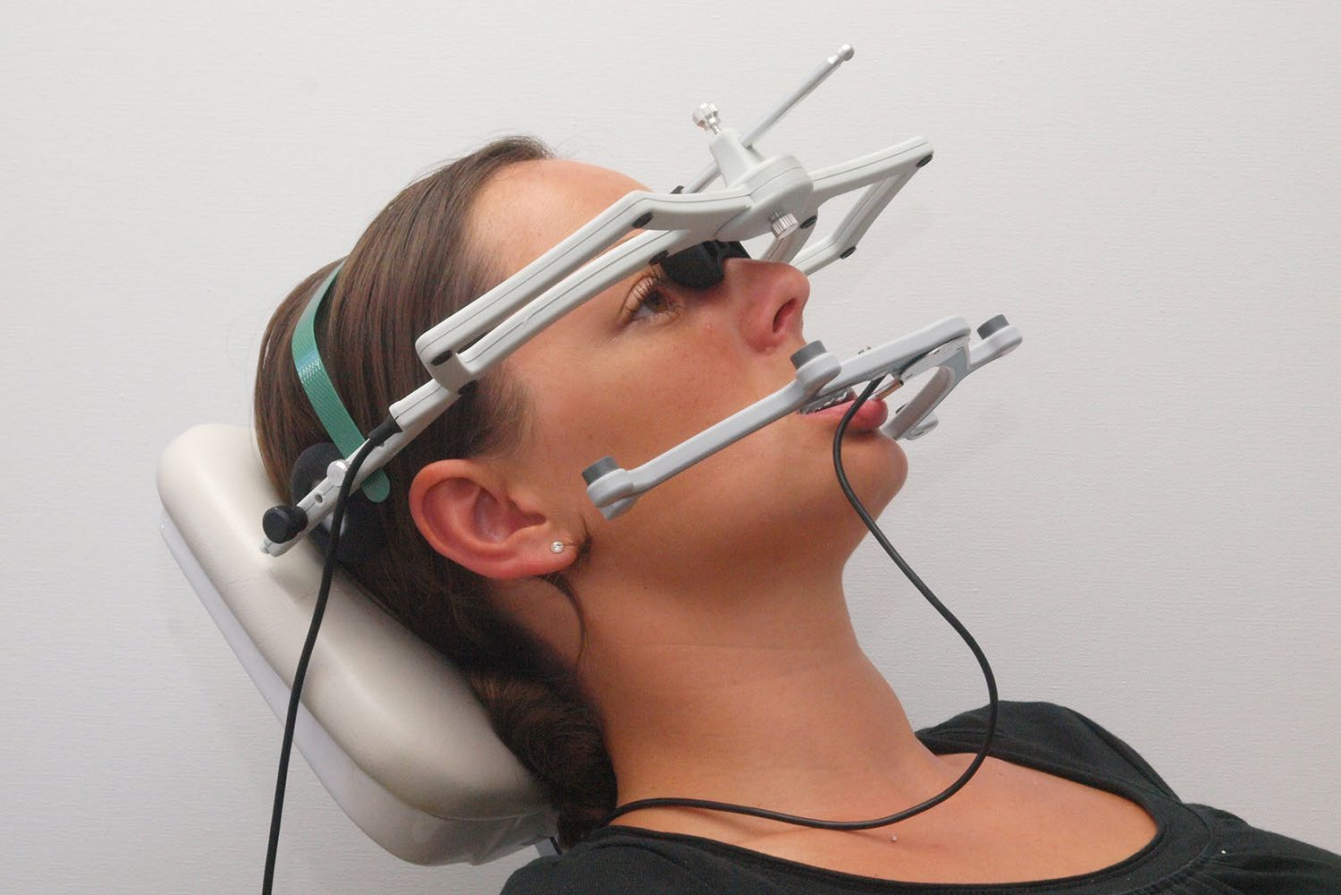
Example of a computer-aided motion recording system based on ultrasonic measurement technology (Zebris Jaw Motion Analyzer+, photo: Ahlers).

Historically, the instruments for recording mandibular motion were initially based on analog recording methods^1^. Computerized methods were not available until the 1970s^2–6^. In the 1990s, electronic recording instruments were introduced for dental practice^7,8^. The earlier recording of movement data on graph paper was replaced by the voltage division method (Fig. 1), ultrasonic measurement technology (Fig. 2), and optoelectronic systems^9^.

As part of articulator programming, devices from all manufacturers use electronically recorded spatial motion data for geometric evaluation. In this process, the condylar/mandibular movement patterns are assigned to the geometries of mechanical articulators and the corresponding setting angles.

Software-based “virtual” articulators have been developed for the CAD/CAM-based production of dental restorations^10–14^. In these virtual articulators, the movement capabilities of mechanical articulators are digitally simulated^7^. For this purpose, the setting values determined from the mandibular motion recording are entered in a user interface for “programming” the selected virtual articulator^15^. The available study results on the quality of these movement simulations revealed good reliability in visualizing dynamic space and contacts, comparable to a mechanic articulator^15–17^, but also determined the heterogeneity of the different analog and digital systems^18^.

Independent of articulator programming, another application has evolved based on the analysis of condylar/mandibular movements. This assessment includes (a) pattern recognition of condylar movements and (b) a velocity curve analysis of condylar movements to assess movement coordination^2,19^.

Pattern recognition of condylar movements dates to the work of Farrar in the 1970s^20,21^. Based on the evaluation of arthrographies, he described the relationship between the timing of TMJ sounds associated with condylar movements and the displacement of the articular disc. Subsequently, other authors included changes in movement velocity and range of motion in the evaluation^22^, which allowed for the assignment of typical movement patterns to individual TMJ dysfunctions^23–28^. This velocity curve analysis of *condylar* movement coordination is based on the evaluation of computerized condylar motion recordings and initially assessed movement capacity^2,29^ and coordination^30,31^. Recently, this has been supplemented by evaluating the movement velocity using computer-assisted path-time diagrams^19^. In evaluating such motion data, a consistent distinction must be made between condylar motion recording and its analysis, as well as systems that do not record condylar movements. This appears important because, for example, for the magnetic kinesiographic systems for neuromuscular motion analysis a study published by Manfredini et al. questioned the validity of kinesiographic recordings in the detection of disc displacements^32^. Hence the German guideline on instrumental functional analysis requests to consequently differentiate between the different systems of motion analysis—*incisal and condylar,* and that condylar movement analysis shall be drawn based on condylar movement registrations^33^. This is supported by Kordaß et al., who determined that when incisal and condylar parameters are compounded, there is a good correlation between computerized measurements of mandibular movements and clinical symptoms of temporomandibular dysfunction^2^. Nevertheless, the diagnosis of temporomandibular disorders in the clinical, but especially in the research context should be mainly based on anamnesis, clinical findings, and imaging (MRI, CT), as there are no internationally accepted criteria for evaluation of condylar motion analysis to date.

The technical prerequisites for these applications are electronic registrations of the condylar/mandibular movements and the possibility of analyzing the recordings later on a PC with the appropriate software. Whereas in articulator programming, only the angular deviation from the reference plane and the curvature of the sagittal and horizontal condylar path are evaluated geometrically, condylar motion analysis assesses spatial motion and changes in movement velocity^19^.

Condylar motion analysis was initially based on uniformly defined instrumental functional findings. These were developed at a consensus conference of the German Society for Functional Diagnosis and Therapy (Deutsche Gesellschaft für Funktionsdiagnostik und -therapie, DGFDT) in 2012 and published internationally^34^. Based on these Criteria, a concept for parameterization and evaluation findings from functional movements analysis was developed^19,35^. The contents of this work have been integrated into the DGFDT guideline on instrumental functional analysis^33^.

Studies on the reproducibility of these evaluations are still a desideratum. Therefore, the present multicenter study aimed to investigate the reproducibility of individual findings for condylar motion analysis parameterized based on the concept mentioned above.

## Material and methods

### Patients

The study was based on the anonymised findings of 20 consecutively recruited patients who presented for diagnosis and treatment of suspected TMD in a center specialized in diagnosing and therapy temporomandibular disorders (TMD) (inclusion criterion). A diagnosis of TMD according to the DC/TMD was not used as an inclusion or exclusion criterion, as the study was not designed to identify TMD patients in this way. Patients were included only if they were older than 18 years and had a sufficiently restored dentition with at least 24 teeth or implants in both jaws. Registrations were made under practice conditions, not for study purposes, but for articulator programming and possible evaluation of condylar movements. A need for further TMD diagnostics was clinically established. All methods were performed in accordance with the relevant guidelines and regulations as stated in the declaration section.

### Instruments

Condylar movements were recorded using the Cadiax compact II system (see Fig. 1) and Gamma Dental Software for Windows, version 8.0.4, module Cadiax Analysis (Gamma Gesellschaft für medizinisch wissenschaftliche Fortbildungen, Klosterneuburg, Austria). All recordings were performed by one single examiner at least twice, as described in the DGFDT guideline^33^.

### Clinical procedure for movement recording

The mandibular bow was attached paraocclusally to the contour of the vestibular surface of the mandibular teeth, bearing electronic transmitters that indicated the condylar movements to the recording devices connected laterally to the maxillary facebow. A procedure published earlier was used, which made it possible to eliminate the need for luting cement or adhesives and to ensure that no material used to attach the mandibular bow covered the occlusal surfaces^36^. To achieve this goal, a paraocclusal adapter (Gamma, see above) was first adapted to the outer contour of the mandibular dentition by pre-bending. Then, an aluminum wax cervical bow was heated and placed on the mandibular arch. The patient was then asked to bite, causing the aluminum wax to cover the occlusal surface. During the subsequent cementation of the paraocclusal adapter with acrylic (Luxabite, DMG-Dental, Hamburg/Germany), no acrylic reached the occlusal surface. After the acrylic had hardened, the aluminum wax half-arch was removed, the patient adopted the habitual occlusion, and the position was recorded as the reference position.

The starting point of the motion recording was the mandibular position currently determined by the habitual occlusion. Movements started from this reference position. The difference between habitual occlusion and centric relation was not assessed in the study, as this information was collected separately by centric relation and habitual occlusion recordings and calculated within the condylar movement analyses. During initial calibration at the beginning of instrumental motion recording, it was noted whether the zero point was maintained, whether a deviation from the zero point was deliberately maintained, or whether the system was recalibrated after identifying a variation. This information is relevant for the dental assessment of the mandibular position after recordings.

Dynamic mandibular movements were performed *unguided,* with and without tooth contact, to determine about the influence of occlusal contacts on the course of condylar/mandibular movement pattern.

Since the condylar movement patterns in the fossa-disc-condylar complex depend on the velocity of the mandibular closing movement and the head posture, the recordings were performed with a physiological lordosis of the cervical spine. For this purpose, the examinations were carried out on a dental chair with a headrest that could be fully adjusted (Finndent FD 7000 patient chair, Finndent Oy, Helsinki, Finland). The back section was set to approximately 120° between the back and the legs. The headrest was adjusted manually to avoid excessive reclination or inclination until patients confirmed their perception of a natural head position. Furthermore, the closing velocity was kept as constant as possible at about 1 Hz^34^; this was previously practiced with the patient. By the specifications of the underlying recommendations^19,35^, deviations of more than 0.3 mm from the starting point were interpreted as unstable.

After the recordings were made, the results obtained were anonymized. For this purpose, the records were copied from the practice data, and the first and last names, as well as the dates of birth, were deleted from the copies. Thus, the records were identified only by a patient number, which could not be traced back to the study participants^36^.

### Investigators

The patients were independently evaluated for the study after a calibration meeting. The prerequisite of the calibration process was the study of the original publication describing the guidelines for the assessment of condylar registration^19^. Based on this, 10 registrations that were not included in the later study were discussed and concluded in a structured Delphi process. In the following, four practitioners analyzed the motion recordings from two specialized institutions in Hamburg (M.O.A., T.P., L.K.) and Leipzig (H.A.J.). The Hamburg-based examiners worked in a Center specializing in TMD diagnosis and therapy from which the data originated. In contrast, the Leipzig-based examiner worked in a university dental clinic where he was responsible for the TMD consultations. The agreement among the examiners' findings was determined by calculating Fleiss' Kappa [κ].

### Evaluation system

**Figure 3:**
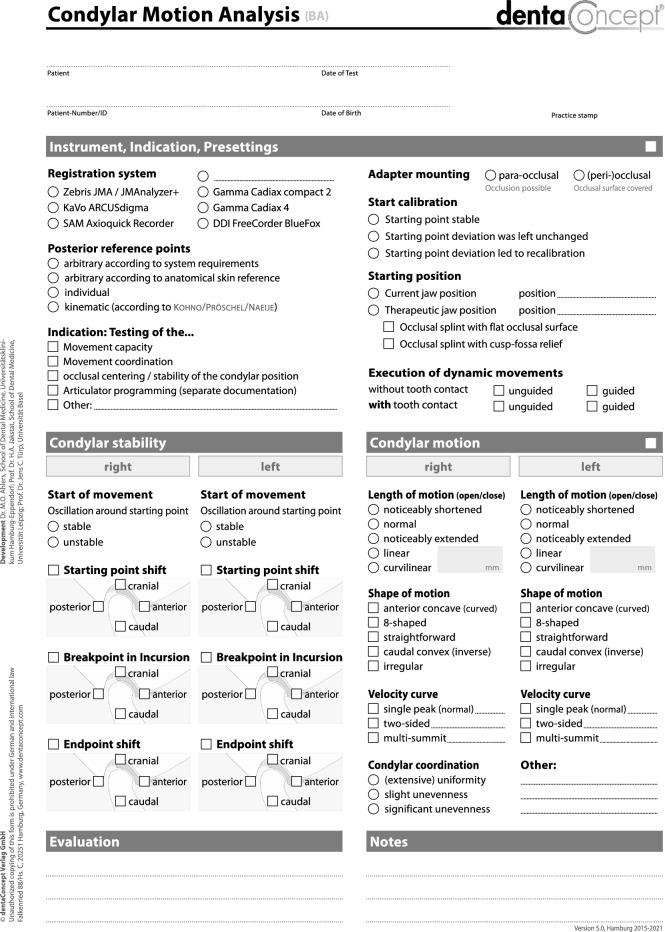
Findings sheet "Instrumental motion analysis" (dentaConcept, Hamburg).

The four examiners evaluated the clinical protocols of all patients. For this purpose, dedicated software was used as the digital counterpart of the “Condylar Motion Analysis" findings sheet (CMDtrace, dentaConcept Publishers, Germany). The software stores the data in an open-source SQL database (Maria DB, Maria DB Foundation, Espoo, Finland (Fig. 3). Each examiner entered their findings into the software on a PC, without access to the data stored by the other examiners.

The previously published evaluation system served as the basis for evaluation^19,35^. The recorded motion data were evaluated within the Cadiax Software on a screen at 100% magnification in specific spatial directions (frontal, sagittal, horizontal). All available recordings were considered; this was done for the right and left TMJ, respectively. The following variables were examined (Table 1).

**Table 1:** Variables examined in the study.

Condylar stability: start/end point displacement
Analysis of the presence of a breakpoint during the closing movement
Condylar motion sequence: path length, path shape
Functional coordination: movement or velocity over time; condylar coordination

#### Basic evaluation data

In the first step, the diagnostic indication for the examination was established. Furthermore, the stored clinical data as well as the recorded condylar motion records were studied, which formed the basis for the subsequent evaluations and findings. These condylar motion records also included information on the registration systems used (see above) and the determination of the posterior reference points. In the further course of this study, the position of the maxillary face-bow with the recording devices attached to it was arbitrarily applied according to the system defaults of the Cadiax Compact 2 registration device.

In case of deviations of the start or end position from the initial calibrated start position during the various recordings, the respective displacement direction of the starting point or end point was determined (cranial, caudal, posterior, anterior), whereby different approaches could be combined (e.g., retral + cranial). The evaluation was primarily based on the jaw *opening curves*, alternatively on the protrusive and retrusive movements with tooth contact.

#### Analysis of condylar stability

**Figure 4:**
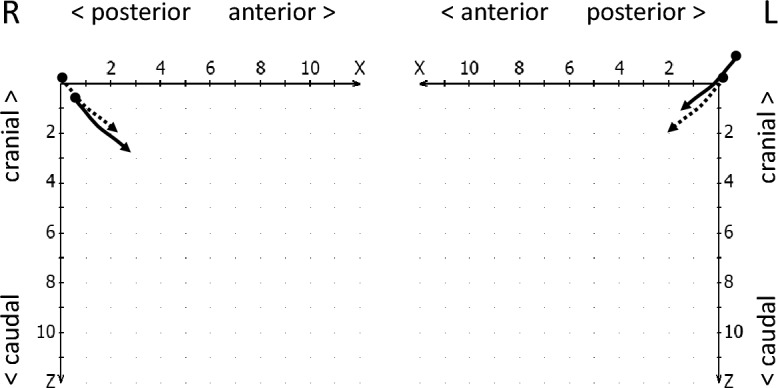
Schematic drawing of a starting point shift to anterior-caudal (right, continous line) and to retro-cranial (left, continous line), both compared to normal movement paths (dotted lines).

**Figure 5:**
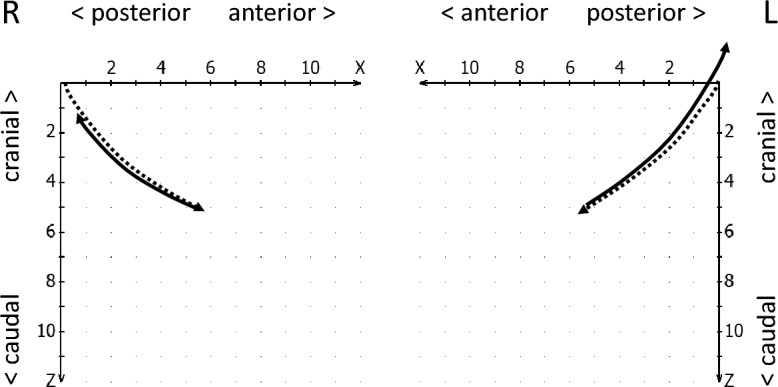
Schematic drawing of an endpoint shift (continuous lines), right to anterior-caudal and left to retro-cranial, as compared to normal movement paths (dotted lines).

**Figure 6:**
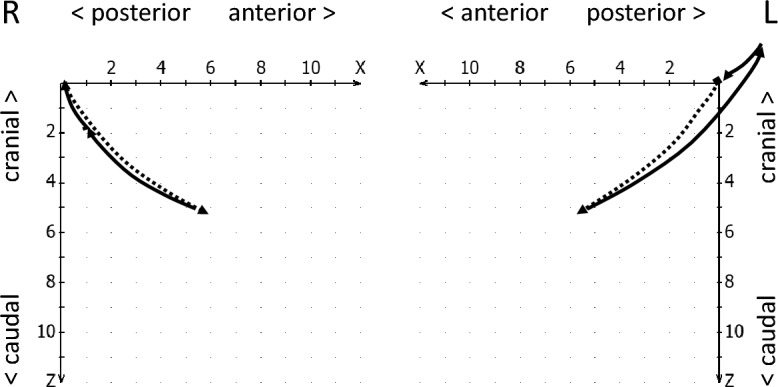
Schematic drawing for a breakpoint in incursion (continuous lines), anterior-caudal on the right, and retro-cranial on the left (as compared to dotted normal movement paths).

The reproducibility of the condylar start and end position in mandibular movements is referred to as condylar stability^19^. Hence, in the condylar stability section, the reproducibility with which the condyles assume the initially calibrated position at the beginning of the movement (starting point shift, Fig. 4) and return to this position at the end of the closing movement (endpoint shift, see Fig. 5) was evaluated. In addition, it was checked whether an intermediate stop in the jaw-closing movement of the condyles (incursion) could be detected^19^, i.e., a short interruption of this movement, whereby the movement velocity increased again (Fig. 6).

#### Analysis of the condylar motion sequence

For the analysis of the condylar motion sequence, the condylar/mandibular movements between the start and end points were parameterized regarding movement capacity and form. For evaluation, the movements opening/closure and protrusion/retrusion were each sampled twice. In case of doubt, the review was based on the non-occlusal guided condylar/mandibular movements in the vertical direction (opening/closure).

**Figure 7:**
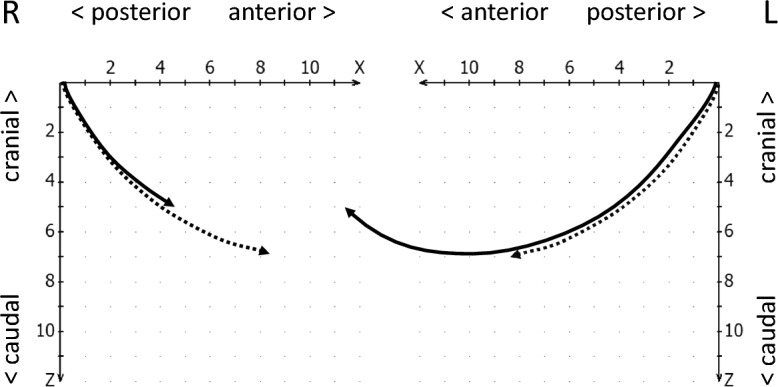
Schematic drawing of a conspicuously shortened movement path (continous line, right side) and a conspicuously lengthened movement path on the left side (normal movement paths dotted, for comparison).

The parameters for evaluating the movement capacity (track/path length) were based on the extent of movement. The classifications relied on corresponding study results^8,29^. Accordingly, the path lengths during opening and closing were classified as follows (Fig. 7):

- Shortening: movement ranges of < 6 mm path length.
- Normal (physiological) length: movement ranges between 6 and 12 mm path length.
- Extension: movement ranges > 12 mm path length.

The movement's most significant deviation from the reference point was the beginning.

**Figure 8:**
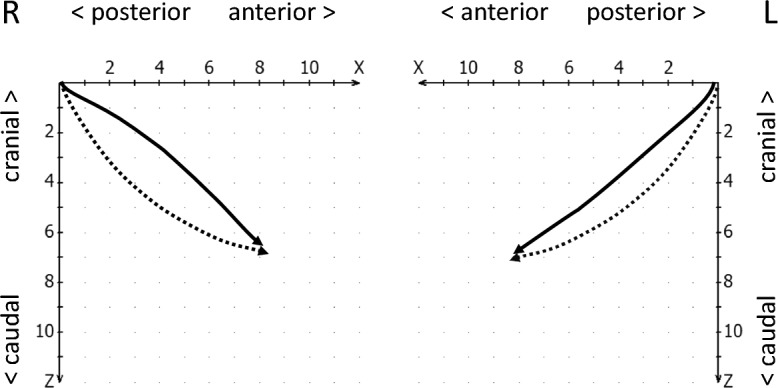
Schematic drawing of caudal convex movement paths (dotted) and anterior concave (continuous line on the right) and straight movement path (continuous line on the left side of the jaw).

**Figure 9:**
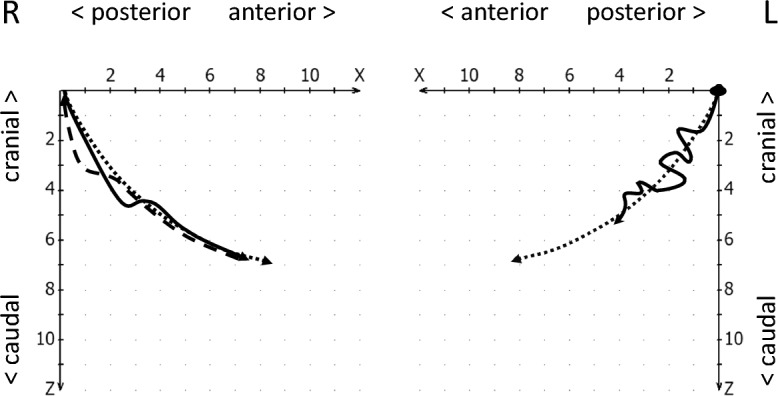
Schematic drawing of an erratic (8-shaped) movement pattern on the right (*excursion* continuous, *incursion* dashed) and an irregular movement pattern (continuous) on the left side of the jaw, both compared to normal caudal convex movement paths (dotted).

The geometry of the movement form (path form) was evaluated using the following feature definitions:

- Concave anterior trajectories are rounded in the caudal direction (Fig. 8, left and right condyles).
- Caudal convex trajectories are rounded cranially (Fig. 8, right condyle).
- Straight condylar trajectories differ from curved trajectories by the curvature index (K) of the trajectory^38^, formed by the length ratio of the secant (d) by the starting and end point of the trajectory, and the maximum deviation of the trajectory from the constructed secant (a). The ratio **κ** = a/d thus describes the curvature of the trajectory, with straight lines characterized by values **κ** < 0.05 (Fig. 8, left condyle).
- Erratic or 8-shaped movements are present when the signal corresponds to the course of a flat number “8” (Fig. 9, right condyle).
- Irregular movements (Fig. 9, left condyle) differ from the previously mentioned possibilities^34^.

#### Analysis of functional coordination

In the “coordination” section of the study, parameterized findings were made concerning the variables “velocity curve” and “condylar coordination.” The results were also based on the criteria previously mentioned^34^ and their design in the published examination and evaluation concept^19,35^.

**Figure 10:**
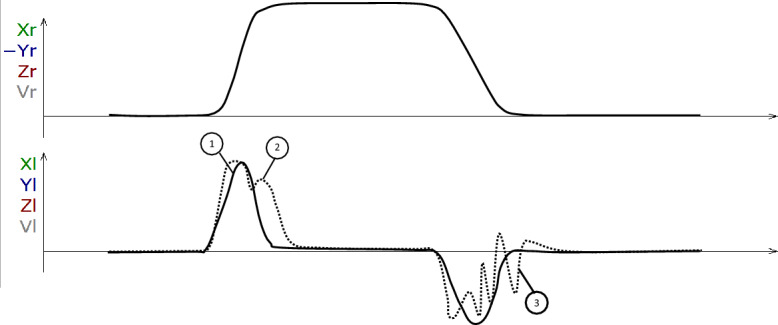
Schematic drawing of a time-distance diagram (upper plot) and a time-speed-diagram (bottom plot) with a single-peaked velocity curve (continuous line marked “1"), a double-peaked velocity curve in the opening phase (dotted line, marked “2") and a multi-peaked velocity curve in the closing phase of the movement (dotted line, marked “3”).

Time-distance diagrams (“time curves”) were used to assess movement velocity over time between start and finish. A second diagram showed the movement velocity at each time along the path (Fig. 10). Based on the shape of the velocity diagram, this was then classified as

- single-peaked,
- two-peaked, or
- multi-peaked.

**Figure 11:**
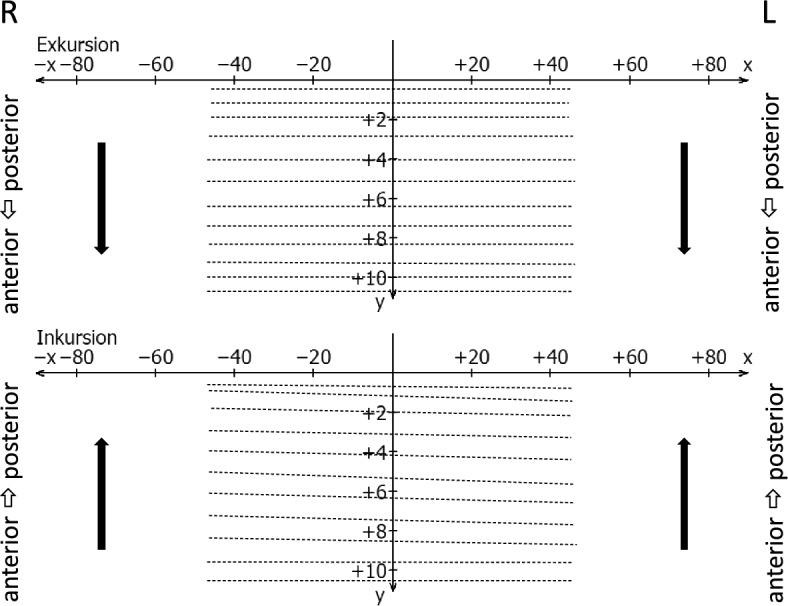
Schematic drawing of essentially uniform condylar coordination during jaw *opening* (excursion, upper plot) and jaw* closing* (incursion, bottom plot) in top view.

Regarding condylar coordination during jaw opening and closure, axial diagrams were first displayed in the top and frontal views as overviews and then viewed twice over time at normal velocity. The line diagrams were examined for parallelism during jaw opening and closure (Fig. 11). Three possibilities were distinguished:

- (extensive) uniformity
- slight unevenness (slight non-parallelism)
- significant unevenness (clear non-parallelism)

**Figure 12:**
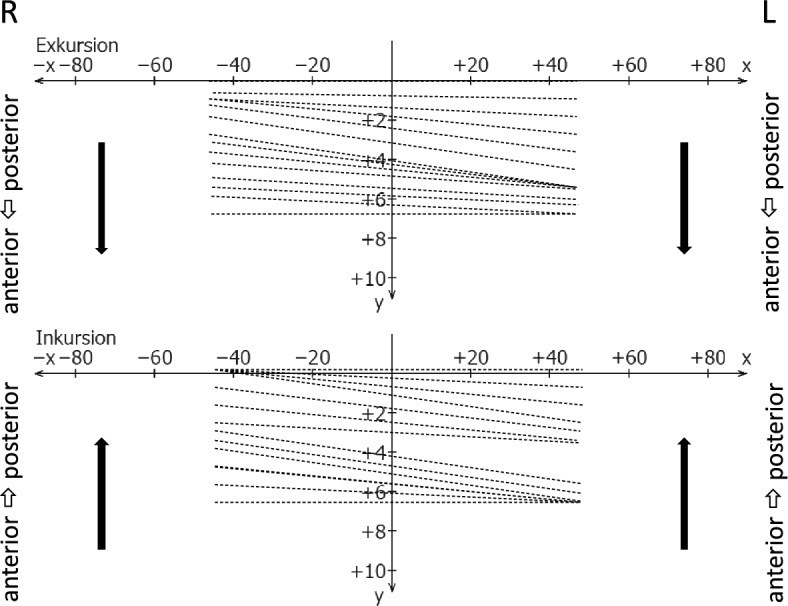
Schematic drawing of an asymmetrical condylar movement with unilateral line compression during jaw opening (excursion, upper plot) and jaw closing (incursion, bottom plot) in horizontal view.

Asymmetries were geometrically represented as line compressions expressing a temporarily lower velocity in one of the two TMJs (Fig. 12).

### Statistical evaluation

For the mathematical evaluation, the collected findings were exported from the database software to Excel (Microsoft 365, Microsoft, Seattle/WA, USA) where they were computed.

The statistical analyses were carried out with the program package SigmaStat 4.0, and the graphical representations of the results were performed with SigmaPlot 13 (Systat Software GmbH, Erkrath, Germany). The data were compared, and the agreement values were determined. Since the data of different raters had to be compared against each other, an extension of the procedure according to Cohen's Kappa was used for several raters (Fleiss' Kappa [κ] method). Hereby, the dichotomous judgments of the raters were tested for agreement. The statement on the quality of the agreement was derived from Fleiss' κ: the more Fleiss' κ approaches 1, the more likely it is that the different investigators will arrive at consistent diagnoses^37^. κ values > 0.4 are considered “moderate agreement”, > 0.6 a “substantial agreement”, and > 0.8 “almost perfect agreement”^38^.

In addition, a test for normal distribution was performed using the Shapiro–Wilk test. Normally distributed data were analyzed parametrically with Student’s t-test. In contrast, non-normally spread data were evaluated non-parametrically with the Mann–Whitney U-test for the presence of significant differences to determine if the agreement among investigators was significantly better than expected by chance (p < 0.05).

#### Null hypothesis

The study was based on the null hypothesis that the examiner’s agreement could be explained by chance alone.

### Ethical approval

The study was performed following the relevant guidelines and regulations and was approved by the Ethics Committee of the Hamburg Medical Association (WF-062/21). The manuscript does not include information or images that could lead to the identification of participants. The study was also conducted following the latest version (2013) of the Declaration of Helsinki of the World Medical Association. Informed consent was obtained from all participants in this study. Written informed consent was obtained from the participant to publish the images in Figs. 1 and 2.

## Results

The evaluations, according to Fleiss’ κ, showed the following results:

### Overall agreement

The overall analysis of all findings across the four examiners yielded a κ of 0.88 (p < 0.00001).

When the evaluation was restricted to the three examiners from the practice, the κ value was 0.91 (p < 0.00001). Both values correspond to an almost perfect agreement. Since these values were so close, only the individual values for all four examiners were subsequently determined (Table 2).

**Table 2:** Overall reproducibility of findings evaluated according to Fleiss’ kappa.

Evaluation	Fleiss’ *κ* (rounded)	p
Overall agreement/3 raters	0.91	< 0.00001
Overall agreement/4 raters	0.88	< 0.00001

### Condylar stability

Evaluation of the agreement in the assessment regarding a starting point shift resulted in a κ***-***value of 0.90 (p < 0.0000001). The agreement regarding the existence of an endpoint shift was even higher (κ = 0.92, p < 0.00001) (Table 3).

**Table 3:** Reproducibility of single findings according to Fleiss' kappa.

Findings	Fleiss’ *κ* (rounded)	p
Starting point	0.90	< 0.0000001
Breaking point	0.67	< 0.00001
Endpoint shift	0.92	< 0.00001
Track/path length	1.00	< 0.0001
Track/path form	0.88	< 0.00001
Velocity curve analysis	0.86	< 0.00001
Functional coordination	0.62	< 0.00001

### Breakpoint in the closing movement

In comparison, an agreement was lower for the detection of a breakpoint (κ = 0.67, p < 0.00001) but still substantial (Table 3).

### Condylar motion path

When evaluating movement capacity (track/path length) for deviations, complete agreement (κ = 1, p < 0.0001) was achieved. The evaluation of the geometry of the movement form (path form) yielded an almost perfect agreement (κ = 0.88, p < 0.00001) (Table 3).

### Functional coordination

The agreement was almost perfect when evaluating the velocity curve (κ = 0.86, p < 0.00001). The evaluation of condylar coordination was more heterogeneous (κ = 0.62, p < 0.00001) but still reached a remarkable agreement (Table 3).

### Refutation of the null hypothesis

The null hypothesis, that the investigators’ agreement could be explained by chance, was refuted in all individual findings.

## Discussion

The general conditions are essential in assessing the significance of the results from this study.

### Experimental design

#### Patients

It should be noted that the movement records were obtained from a specialized center for TMD diagnosis and management, which almost exclusively cares for patients who could not be successfully managed elsewhere and were therefore referred. Hence, more abnormal findings were expected in this patient population compared to patients in an average dental office. However, for this investigation—to examine the reproducibility of the diagnostic conclusions—patients with an exceptionally high number of clinical signs were helpful.

The study’s results are externally valid because patients were not purposively selected but were recruited consecutively without exception, and the motion recordings were made under practice conditions. Accordingly, a control group was not necessary as the aim of the study was to test the reliability of the criteria presented. Based on this information, future studies could determine whether the criteria are more or less common in different subgroups of TMD patients.

#### Investigators

The four examiners were specialized dentists with many years of expertise in evaluating condylar motion recordings. This is not a limitation of the study but was an intended requirement to ensure that the study results reflected the findings’ reproducibility rather than differences in the qualifications of the investigators.

However, as with all skill-based examinations and techniques, dentists with less experience may produce more heterogeneous results, with a higher proportion of noise and bias.

The selection of the four examiners from two locations, with distribution among three examiners working closely together at one place and one examiner who had no contact with the other examiners in practice, was designed to show whether regular cooperation and coordination strongly influenced outcomes. The results refuted this hypothesis; there was little difference among the scores.

#### Instruments

The computerized registration system used in the study was selected because it has been in practice for many years, the team is well trained in the use of the system and because the associated software (Cadiax Analysis) fully enables the display of condylar/mandibular movements for functional movement analysis, if the user interface is appropriately set. The decision to use the Gamma Cadiax compact 2, which is based on an arbitrary reference point, was made in order to minimise the examination technique and to focus the evaluation on the movement trajectory rather than the deviation of the kinematic hinge axis from the condylar position in habitual occlusion. Nevertheless, the more complex para-occlusal fixation of the mandibular arch was deliberately chosen to allow for uninterrupted adjustment of the mandible in habitual occlusion.

The analysis system is completely manufacturer-independent^19,35^. Thus, other computerized condylar registration systems that meet these requirements could have been used—especially since different systems ensure the technical and diagnostic validity and reliability of the motion data with their signal quality^23^.

### Evaluation system

The evaluation system is based on consensus-based criteria to evaluate instrumental registrations^34^. The study aimed to test the suitability of the evaluation system for use with the diagnostic reporting system employed here. The high agreement values have confirmed this suitability.

This is also true for the starting point, although interpreting a starting point that deviates more than 0.3 mm from the reference point as “unstable” may seem like a very small cut-off point—since even habitual intercuspidation can result in such minimal deviation. Condylar movement, on the other hand, usually starts from this position. In addition, the recordings were made using paraocclusal registration, which allows patients to control their mandibular position with the aid of occlusion perception.

#### Areas with conspicuously lower agreement

Given the overall high agreement, two values were conspicuously lower in comparison:

- Analysis of condylar stability: break in the closing movement (incursion). This was defined as a short interruption of a closing movement during which the motion velocity increased again. In TMJ arthropathies, this course may indicate that a physiological TMJ position is adopted that prevents dysfunctional displacement, especially in the retral and cranial direction^39^.
- Analysis of functional condylar coordination. Here, a review of the causes of the comparatively low agreement showed that the definition is open to different interpretations. A distinction should be made between no, slight, and marked nonparallelism, whereby the mandibular opening and closure findings were recorded together.

#### Definition of the criteria and adjustment of the finding’s matrix

Because of the results, the differently interpreted records were reevaluated—this time openly—after the completion of the study evaluation. This new analysis revealed misunderstandings in the definition of the respective findings as the cause of the selectively lower agreements. The following adjustments are therefore necessary for improvement:

- By means of a structured Delphi process we therefore developed a refinement of the definition. Based on this refinement in a reevaluation of the data we reached a high Fleiss κ of 0.9 (p < 0.05). Therefore, we propose to define a stopping not as a *point* where the movement stops absolutely with a sudden velocity drop towards zero but as an *area* where the condyle in question slows down abruptly during the mandibular closing movement with a noticeable velocity drop, followed by a subsequent increase of the movement velocity.
- Furthermore, we advocate distinguishing only between even or asymmetrical/nonparallel in condyle coordination. For more transparency, each finding is recorded separately for jaw opening and closing. This avoids inconsistent evaluations when combining these two condylar/mandibular movements.

In addition, the available evaluation revealed the following possible sources of ambiguous findings:

- Multiple selections were previously possible when evaluating trajectory shape. This did not lead to an improvement in the 20 patients examined. Therefore, only *one* option should be selectable.
- Multiple selections were also previously possible for the movement velocity findings to be able to image different states of jaw opening and closure. Here, too, only one option should be selectable.

### Classification of the values

Other studies on the reproducibility of findings in instrumental motion analysis examined the reproducibility of trajectories in registration systems with arbitrary and kinematic hinge axis localization. They achieved an intra-class correlation (ICC) of > 0.8 for the Cadiax compact II registration system, also used in our study. They found strong similarities in the characteristics of the motion paths^8^. Comparable data were obtained from a multicenter study with five trained and calibrated examiners using the JMA ultrasound measuring instrument (Zebris Medical) for sagittal condylar track inclination (ICC 0.87–0.91) and sagittal and lateral anterior guidance (ICC 0.88–0.99). This reproducibility of unguided and guided Bennett movement alone was significantly lower (0.44–0.62)^40^. Our results complement these findings with the values for functional motion analysis and show that a similarly high reproducibility was achieved with the evaluation system used.

In clinical functional analysis, corresponding investigations were performed for the Diagnostic Criteria for Temporomandibular Disorders (DC/TMD)^41^. The reliability achieved in the clinical evaluation in connection with the DC/TMD criteria for pain related TMDs was excellent, with a κ value ≥ 0.85^41^. The agreements in this study, apart from the two exceptions mentioned above, were of the same order of magnitude and, in some cases, even higher, indicating that the system developed for the evaluation of instrumental functional motion analysis meets the requirements of functional analysis.

### Perspectives

#### …for the practice

The results determine that the condylar motion analysis is a reliable tool for functional analysis, at least for dentists trained in this technique, and helps evaluate clinical situations based on the findings.

#### … for science

In the future, it would also be helpful to repeat the present study on the reliability of the findings with trained, but clinically inexperienced investigators. This would allow testing the extent to which less experienced examiners can correctly assess the results of functional movement analysis and determine if the criteria are also useful in general dental practice.

If appropriate criteria are applied, this type of motion analysis evaluation appears to have a high degree of reliability. This serves as the prerequisite for further validation of these criteria regarding internationally comparable criteria (DC/TMD).

## Data Availability

The original raw data was uploaded along with the paper as related files and are thus accessible. Additional materials are available upon request from the authors by contacting the Corresponding author.
